# Psychosocial work exposures as risk factors for skin problems in a general working population: cross-sectional and prospective associations

**DOI:** 10.1007/s00420-025-02135-w

**Published:** 2025-03-11

**Authors:** Randi Hovden Borge, Håkon A. Johannessen, Jose Hernán Alfonso

**Affiliations:** 1 Department of Work Psychology and Physiology, National Institute of Occupational Health, Pb 5330 Majorstuen, 0304 Oslo, Norway; 2 Department of Occupational Medicine and Epidemiology, National Institute of Occupational Health, Oslo, Norway; 3 Department of Dermatology and Venereology, Oslo University Hospital, Rikshospitalet, Oslo, Norway

**Keywords:** Skin disease, Psychosocial work environment, Work stress, Occupational dermatology, Epidemiology, General working population

## Abstract

**Objective:**

The potential contribution of psychosocial work exposures to skin problems is largely overlooked in the occupational health literature. To address this knowledge gap, we examined cross-sectional and prospective associations between six psychosocial work exposures (i.e., quantitative demands, job control, social support, emotional demands, role conflict, and interpersonal conflict) and self-reported skin problems.

**Methods:**

Data came from a probability sample of the general working population in Norway surveyed in 2016 (N = 7833) and 2019 (N = 8038). The prospective sample comprised 3430 participants. Data were analysed with ordered logistic regression, adjusting for age, sex, occupation, and exposure to cleaning products, water, and dry indoor air.

**Results:**

Cross-sectional analyses indicated statistically significant associations with skin problems for emotional demands, role conflict, and interpersonal conflict in 2016 and 2019, and for social support in 2019. In prospective analyses, emotional demands (OR 1.12, 95% CI 1.01–1.23), role conflict (OR 1.14, 95% CI 1.00–1.29), and interpersonal conflict (OR 1.24, 95% CI 1.01–1.52) significantly predicted subsequent skin problems. Interpersonal conflict (OR 1.26, 95% CI 1.01–1.57) was a significant predictor above and beyond baseline levels of skin problems. Quantitative demands and job control was generally non-significant, except for a significant interaction in the 2019 sample.

**Conclusion:**

Exposure to certain psychosocial work stressors may be a risk factor for experiencing skin problems, particularly if you are exposed to interpersonal conflict. Preventive efforts to reduce the occurrence of skin problems in work settings should also target psychosocial stressors.

**Supplementary Information:**

The online version contains supplementary material available at 10.1007/s00420-025-02135-w.

Skin problems are among the most common health complaints worldwide and represent a considerable public and occupational health concern (Flohr and Hay 2021; Richard et al. 2022). Therefore, population-based studies assessing work-related risk factors for skin problems are necessary for effective surveillance and primary prevention of skin problems in the general working population. While the role of chemical and physical work exposures in the development and worsening of skin problems is well known (Alfonso et al. 2015), the potential contribution of psychosocial work exposures has largely been overlooked in the scientific literature. For instance, skin problems were not among the many outcomes featured in a recent meta-review of research on psychosocial work exposures and a broad range of physical and mental health complaints (Niedhammer, Bertrais and Witt, 2021). This represents a clear gap in the occupational health literature considering many skin conditions are thought to be related to psychophysiological stress responses (Kimyai-Asadi and Usman 2001; Alexopoulos and Chrousos 2016; Goyal and Prabhu 2023; Zhang et al. 2024). Synthesized evidence also suggests significant associations between symptoms of depression and anxiety, which are common outcomes of repeated or chronic exposure to psychosocial work stressors (Harvey et al. 2017), and common skin diseases such as atopic dermatitis (Rønnstad et al. 2018). Studies that investigate the empirical linkages between psychosocial work exposures and skin problems therefore seem overdue.

Psychosocial work exposures may contribute to the development or worsening of skin problems via adaptive systems activated in the body and brain in response to situations perceived as challenging or threatening (Nixon et al. 2011; Zhang et al. 2024). A dominant framework for explaining how these processes may lead to disease over time is the allostatic load model (McEwen and Stellar 1993; McEwen 1998), centring around the concepts of allostasis and allostatic load. Allostasis refers to the activation of important neural, neuroendocrine, and neuroendocrine-immune mechanisms that enable the body to cope and adapt to environmental challenge. While essential to our everyday functioning and survival, allostasis is taxing in the long term. Allostatic load refers to the cumulative burden on the body and brain resulting from repeated or chronic activation of the adaptive systems involved in allostasis, for instance if we are frequently or heavily exposed to psychosocial stressors at work (Nixon et al. 2011; Ganster and Rosen 2013). Persisting over time, allostatic load may predispose individuals to disease (Juster, McEwen and Lupien, 2010). The skin, the largest body organ, is part of the body’s overall stress response system (Alexopoulos and Chrousos 2016; Zhang et al. 2024), and allostatic load may play a significant role in the development and worsening of common skin diseases like atopic dermatitis and psoriasis (Alexopoulos and Chrousos 2016; Goyal and Prabhu 2023; Zhang et al. 2024). Moreover, psychophysiological stress negatively impacts skin barrier function in both murine models (Denda et al. 1998; Denda et al. 2000) and humans (Garg et al. 2001) without previous skin disease. Corroborating the above, patients with hand dermatoses often believe that stress negatively influences their skin disease (Niemeier et al. 2002).

Based on the above reasoning, exposure to psychosocial work stressors represents a potential risk factor for skin problems that warrants attention. The paucity of occupational health research investigating these linkages, particularly in general working populations, is therefore striking (for exemption studies in specific occupational groups, see Japundžić et al. (2023), Magnavita et al. (2011), Onder et al. (1999), and Eriksson (1997)). To address this clear knowledge gap, we examined cross-sectional and prospective associations between six distinct psychosocial work exposures (i.e., quantitative demands, job control, social support, emotional demands, role conflict, and interpersonal conflict) and self-reported skin problems using data from a large probability sample of the general working population in Norway. Quantitative demands, job control, and social support were chosen based on the demand-control-support model (Karasek 1979; Johnson and Hall 1988). In addition to main effects of quantitative demands and job control on employee health and well-being, the model depicts that high job strain (i.e., high demands accompanied by low control) will lead to stronger stress reactions, especially in case of low social support (Häusser et al. 2010). We therefore also examined two-way and three-way interactions between quantitative demands, job control, and social support on skin problems.

## Methods

### Sample and procedure

The current study used data from two consecutive survey waves conducted in 2016 and 2019 as part of the Level of Living Surveys on Working Conditions (Statistics Norway, 2022; LKU-A). The LKU-A is a nationally representative survey conducted every third year that tracks a wide range of occupational exposures and employee outcomes in the Norwegian working population (Revold and Bye 2017). The LKU-A was conducted for the first time in 2006, when a random sample of 18 999 individuals from the Norwegian population between 17 and 67 years of age was invited to participate. The same set of individuals was invited to participate in the two consecutive waves (i.e., 2009 and 2013). From 2016 onwards, the LKU-A operates according to a rotation sampling method across survey waves. In 2016, the original sample was divided into three equal groups, out of which two groups were retained, and one was replaced by a new set of randomly drawn individuals who had not previously participated. The same procedure was performed in 2019; one of the two remaining groups from the 2006 sample was retained and the other was replaced by a new group of randomly drawn individuals.

Survey interviews were conducted by personal telephone between September 2016 and April 2017 (wave 1) and October 2019 and March 2020 (wave 2). Potential participants received written information by mail prior to telephone contact and participation was based on informed consent. The response rates were 53% in 2016 (10,665 participated out of 20,272 invited) and 57% in 2019 (11,212 out of 20,272 invited). Eligible participants for each of the cross-sectional samples were employees in paid work when the survey was conducted. Eligible participants for the prospective sample were those who were invited and responded to the survey in both 2016 and 2019 and reported being in paid work at both timepoints. Participants who were self-employed or sole owner of their own company were excluded from all three samples because they had not received questions about several of the psychosocial work factors (e.g., job control, interpersonal conflict). Final samples consisted of 7833 participants in 2016, 8038 in 2019, and 3430 in the prospective sample.

The rotation sampling procedure described above, in addition to dropout between survey waves, resulted in a smaller prospective sample compared to the two cross-sectional samples. The attrition due to the rotation sampling procedure is random by design (Revold and Bye 2017). Attrition analyses based on those who were invited back for the 2019 survey (i.e., looking only at dropout due to non-response) showed that none of the study variables significantly predicted non-response in 2019 after adjusting for demographic covariates. The log-odds of non-response in 2019 was slightly higher for women than for men (0.025, p < 0.05) and for several occupations compared to professionals (clerical support 0.102, p < 0.001; service and sales 0.114, p < 0.001; craft and related trades 0.058, p < 0.05; plant and machine operators 0.106, p < 0.001; others 0.096, p < 0.01). Yet, overall, the variables explained very little variance in non-response (R2_McFadden_ = 0.009).

### Exposure measurement

*Quantitative demands* were measured by two questions, addressing work pace and workload, respectively. Both items came from the General Nordic Questionnaire for Psychological and Social Factors at Work (Dallner et al. 2000). Participants responded on a scale from 1 (“very seldom or never”) to 5 (“very often or always”). The two items were combined into a mean score with high scores representing high demands. Scale reliability (Cronbach’s alpha; α) was 0.71 in 2016 and 0.72 in 2019.

*Job control* was measured by four questions addressing control over (i) timing at work (one item), (ii) methods at work (two items), and (iii) decisions at work (one item). The first item addressing timing control and the fourth item addressing decision control came from the General Nordic Questionnaire for Psychological and Social Factors at Work (Dallner et al. 2000). The two remaining items addressing method control were developed for use in the LKU-A by Statistics Norway (Revold and Bye 2017). Participants responded on a scale from 1 (“to a very high extent”) to 5 (“to a very small extent”). The four items were reverse coded and combined into a mean score with high scores representing high levels of job control (α = 0.76/0.75).

*Social support* was measured by two questions capturing support from (i) colleagues and (ii) immediate supervisor. Both items came from the General Nordic Questionnaire for Psychological and Social Factors at Work (Dallner et al. 2000). Participants responded on a scale from 1 (“very seldom or never”) to 5 (“very often or always”). The two items were combined into a mean score with high scores representing high levels of social support (α = 0.57/0.56).

*Emotional demands* were measured by two questions addressing the extent to which participants had to (i) hide own negative emotions or (ii) deal with such emotions as part of their work with customers, clients, or others outside their organization. Both items were developed for use in the LKU-A by Statistics Norway (Revold and Bye 2017). Participants responded on a scale from 1 (“to a very high extent”) and 5 (“not at all”). Both items were reverse coded before combined into a mean score with high scores representing high emotional demands (α = 0.70/0.71).

*Role conflict* was measured by three questions addressing how often participants: (i) performed tasks that they thought should be performed differently, (ii) performed tasks without necessary equipment or resources, or (iii) received conflicting requests from two or several people. All items came from the General Nordic Questionnaire for Psychological and Social Factors at Work (Dallner et al. 2000). Participants responded on a scale from 1 (“very seldom or never”) to 5 (“very often or always”). The three items were combined into a mean score with high scores representing high levels of role conflict (α = 0.68/0.68).

*Interpersonal conflict* was measured by five questions addressing how often participants experienced: (i) poor social relations between management and employees, (ii) poor social relations between employees, (iii) uncomfortable conflicts with superiors, (iv) uncomfortable conflicts with colleagues, and (v) uncomfortable conflicts with customers, clients, or others outside their organization. All items were developed for use in the LKU-A by Statistics Norway (Revold and Bye 2017). Participants responded on a scale from 1 (“often”) to 4 (“never”). All items were reverse coded before combined into a mean score with high scores representing high levels of interpersonal conflict (α = 0.72/0.71).

### Outcome measurement

*Skin problems* were an ordered categorical variable with four levels (i.e., “not afflicted”, “a little afflicted”, “somewhat afflicted”, “severely afflicted”) based on the question: “have you over the past month been severely afflicted, somewhat afflicted, a little afflicted, or not afflicted by eczema, itchy skin, or rash?”. The question was developed by Statistics Norway (Revold and Bye 2017).

### Confounding and adjustment variables

The exposure to and experience of psychosocial work exposures may depend on demographic characteristics and type of work. The same characteristics may impact the occurrence and reporting of skin problems (Alfonso et al. 2015). We therefore adjusted for age (in years), sex (male or female), and main occupation group (dummy variables based on the International Standard Classification of Occupations (ISCO). We also adjusted for exposure to water, cleaning products, and indoor dry air, as these have been found to significantly predict skin problems in previous studies in the Norwegian working population (Alfonso et al. 2015). All demographic variables came from registry data. Exposure to water, cleaning products, and indoor dry air was self-reported (i.e., “are you in your daily work exposed to […]” to which participants answered “yes” or “no”). In the prospective analyses, we ran separate models where we also adjusted for levels of skin problems in 2016 as a linear continuous predictor.

### Statistical analysis

We prepared and analysed data in R version 4.3.2 (R Core Team 2023). We estimated ordered logistic regression models with the clm() function from the ordinal package (Christensen 2023). Estimated effects are reported in odds ratios (OR) and 95% confidence intervals (CI). Statistical significance was determined by a significance level of P < 0.05. Odds ratios in an ordered logistic regression model represents the effect of a one unit increase in a predictor on falling into or below any response category of the ordinal outcome variable. Each of the psychosocial work exposures were tested separately. Unadjusted models (model 1) included the psychosocial factor as the sole predictor. Model 2 was adjusted for demographic characteristics. Model 3 also included exposure to water, cleaning products, and indoor dry air. Model 4 in the prospective analyses included all variables above, plus skin problems measured in 2016. According to the Brant-Wald test (Brant 1990), the proportional odds assumption was satisfied for all the psychosocial work exposures (quantitative demands: *X*^2^(2) = 3.86, P = 0.14; job control: *X*^2^(2) = 2.38, P = 0.3; social support: *X*^2^(2) = 1.81, P = 0.41; emotional demands: *X*^2^(2) = 3.13, P = 0.21; role conflict: *X*^2^(2) = 2.05, P = 0.36; interpersonal conflict: *X*^2^(2) = 2.02, P = 0.36).

Two-way and three-way interactions between quantitative demands, job control, and social support were tested by adding multiplicative terms involving the respective variables to fully adjusted models. These models were compared against models without multiplicative terms with chi-squared difference tests (i.e., omnibus tests of interaction effects). The psychosocial work factors were centred at the mean before entered into the model. In case of a significant chi-squared difference test, the interaction was further probed.

## Results

Table 1 displays characteristics of the 2016, 2019, and prospective samples. Prevalence of skin problems were between 12 and 13 percent in all three samples.

**Table 1:** Characteristics of the study samples

	Prospective sample	2016 sample	2019 sample
Characteristic	N = 3,430^*1*^	N = 7,833^*1*^	N = 8,038^*1*^
Skin problems in 2016			
Not afflicted	3,010 (88%)	6,883 (88%)	
A little afflicted	297 (8.7%)	679 (8.7%)	
Somewhat afflicted	83 (2.4%)	195 (2.5%)	
Severely afflicted	31 (0.9%)	76 (1.0%)	
(missing)	9		
Skin problems in 2019			
Not afflicted	3,014 (88%)		7,009 (87%)
A little afflicted	314 (9.2%)		763 (9.5%)
Somewhat afflicted	76 (2.2%)		187 (2.3%)
Severely afflicted	26 (0.8%)		79 (1.0%)
Quantitative demands in 2016	3.58 (0.92)	3.53 (0.94)	
(missing)	15	35	
Quantitative demands in 2019	3.62 (0.94)		3.56 (0.97)
(missing)	5		25
Job control in 2016	3.44 (0.80)	3.43 (0.83)	
(missing)	29	65	
Job control in 2019	3.44 (0.82)		3.56 (0.82)
(missing)	24		87
Social support in 2016	4.17 (0.87)	4.15 (0.89)	
(missing)	47	149	
Social support in 2019	4.16 (0.85)		4.19 (0.86)
(missing)	32		85
Emotional demands in 2016	2.39 (1.06)	2.39 (1.07)	
(missing)	29	61	
Emotional demands in 2019	2.34 (1.03)		2.33 (1.06)
(missing)	26		70
Role conflict in 2016	2.14 (0.81)	2.11 (0.82)	
(missing)	35	97	
Role conflict in 2019	2.23 (0.84)		2.19 (0.85)
(missing)	34		119
Interpersonal conflict in 2016	1.82 (0.51)	1.79 (0.53)	
(missing)	54	135	
Interpersonal conflict in 2019	1.83 (0.51)		1.79 (0.53)
(missing)	51		119
Sex			
Men	1,825 (53%)	4,089 (52%)	4,217 (52%)
Women	1,605 (47%)	3,744 (48%)	3,821 (48%)
Age	43 (12)	43 (13)	42 (13)
Occupation			
Managers	380 (11%)	830 (11%)	826 (10%)
Professionals	1,255 (37%)	2,597 (33%)	2,675 (33%)
Technicians	615 (18%)	1,282 (16%)	1,377 (17%)
Clerical support	168 (4.9%)	441 (5.6%)	422 (5.3%)
Service and sales	500 (15%)	1,374 (18%)	1,415 (18%)
Craft and related trades	253 (7.4%)	604 (7.7%)	672 (8.4%)
Plant and machine operators	138 (4.0%)	384 (4.9%)	380 (4.7%)
Others	121 (3.5%)	321 (4.1%)	271 (3.4%)
Exposed to water	509 (15%)	1,306 (17%)	1,432 (18%)
(missing)	13	29	17
Exposed to cleaning products	346 (10%)	867 (11%)	913 (11%)
(missing)	8	23	4
Exposed to dry indoor air	322 (9.4%)	774 (9.9%)	916 (11%)
(missing)	10	25	9
^*1*^ n (%); Mean (SD)			

### Cross-sectional associations

Results from the two cross-sectional analyses (Tables 2 and 3) indicated statistically significant associations with skin problems for emotional demands, role conflict, and interpersonal conflict in both 2016 and 2019 and for social support in 2019, also after adjusting for age, sex, occupation, and exposure to cleaning products, water, and dry indoor air. The strongest association was for interpersonal conflict in the 2019 sample. The association between job control and skin problems was significant in unadjusted and partly adjusted models, but turned non-significant after fully adjusting for covariates (model 3). The association between quantitative demands and skin problems was largely non-significant, apart from in unadjusted and partly adjusted models in 2019.

**Table 2:** Cross-sectional associations between psychosocial work factors and skin problems in 2016. Odds ratios (OR) and 95% confidence intervals (95% CI). Statistically significant associations in **bold**

	Model 1^*a*^		Model 2^*b*^		Model 3^*c*^	
Factor	OR	95% CI	OR	95% CI	OR	95% CI
Quantitative demands	1.03	0.95–1.10	1.02	0.95–1.10	1.00	0.93–1.08
Job control	**0.88**	0.81–0.95	**0.90**	0.82–0.98	0.92	0.84–1.01
Social support	1.02	0.94–1.10	1.01	0.93–1.09	1.02	0.94–1.10
Emotional demands	**1.19**	1.11–1.26	**1.16**	1.08–1.23	**1.14**	1.06–1.21
Role conflict	**1.18**	1.09–1.28	**1.17**	1.08–1.27	**1.15**	1.06–1.25
Interpersonal conflict	**1.32**	1.17–1.50	**1.30**	1.15–1.48	**1.26**	1.11–1.43

^*a*^Unadjusted model

^*b*^Adjusted for age, sex, and occupation

^*c*^Adjusted for age, sex, occupation, and exposure to cleaning products, water or dry indoor air in 2016

**Table 3:** Cross-sectional associations between psychosocial work factors and skin problems in 2019. Odds ratios (OR) and 95% confidence intervals (95% CI). Statistically significant associations in **bold**

	Model 1^*a*^		Model 2^*b*^		Model 3^*c*^	
Factor	OR	95% CI	OR	95% CI	OR	95% CI
Quantitative demands	**1.08**	1.01–1.15	**1.09**	1.01–1.17	1.06	0.99–1.13
Job control	**0.88**	0.81–0.95	**0.91**	0.83–0.99	0.94	0.86–1.02
Social support	**0.89**	0.83–0.96	**0.88**	0.82–0.95	**0.90**	0.83–0.97
Emotional demands	**1.17**	1.10–1.24	**1.13**	1.06–1.20	**1.08**	1.01–1.15
Role conflict	**1.21**	1.12–1.31	**1.22**	1.13–1.32	**1.17**	1.08–1.26
Interpersonal conflict	**1.57**	1.39–1.78	**1.58**	1.40–1.79	**1.47**	1.30–1.67

^*a*^Unadjusted model

^*b*^Adjusted for age, sex, and occupation

^*c*^Adjusted for age, sex, occupation, and exposure to cleaning products, water or dry indoor air in 2019

### Prospective associations

Table 4 displays results from the prospective analyses. Emotional demands, role conflict, and interpersonal conflict in 2016 significantly predicted skin problems in 2019 in unadjusted models and after adjusting for age, sex, occupation, and exposure to cleaning products, water, and dry indoor air. Interpersonal conflict remained statistically significant also after adjusting for baseline levels of skin problems. Quantitative demands, job control, and social support in 2016 did not significantly predict skin problems in 2019.

**Table 4:** Prospective associations between psychosocial work factors and skin problems. Odds ratios (OR) and 95% confidence intervals (95% CI). Statistically significant associations in **bold**

	Model 1^*a*^		Model 2^*b*^		Model 3^*c*^		Model 4^*d*^	
Factor	OR	95% CI	OR	95% CI	OR	95% CI	OR	95% CI
Quantitative demands	0.95	0.85–1.06	0.97	0.87–1.09	0.96	0.85–1.07	0.97	0.86–1.10
Job control	0.92	0.81–1.04	0.95	0.83–1.09	0.99	0.86–1.13	0.98	0.85–1.13
Social support	0.93	0.83–1.05	0.92	0.82–1.04	0.93	0.83–1.05	0.91	0.80–1.03
Emotional demands	**1.16**	1.05–1.27	**1.14**	1.03–1.26	**1.11**	1.01–1.23	1.09	0.97–1.21
Role conflict	**1.16**	1.02–1.32	**1.17**	1.03–1.33	**1.14**	1.00–1.29	1.10	0.96–1.26
Interpersonal conflict	**1.28**	1.05–1.56	**1.30**	1.07–1.59	**1.24**	1.01–1.52	**1.26**	1.01–1.57

^*a*^Unadjusted model

^*b*^Adjusted for age, sex, and occupation

^*c*^Adjusted for age, sex, occupation, and exposure to cleaning products, water or dry indoor air in 2016

^*d*^Adjusted for age, sex, occupation, exposure to cleaning products, water or dry indoor air in 2016, and skin problems in 2016

### Demand-control-support interactions

Omnibus tests of interactions between quantitative demands, job control, and social support indicated a statistically significant two-way interaction between quantitative demands and job control in the expected direction in the 2019 sample (*X*^*2*^(1) = 4.039, P = 0.045), but not in the 2016 sample (*X*^*2*^(1) = 0.506, P = 0.477) or the prospective sample (*X*^*2*^(1) = 0.286, P = 0.593). The three-way interaction with social support was not significant in any of the samples (2016 sample: *X*^*2*^(3) = 0.229, P = 0.973; 2019 sample: *X*^*2*^(3) = 2.543, P = 0.467; prospective sample: *X*^*2*^(3) = 2.089, P = 0.554). Probing of the significant two-way interaction in the 2019 sample indicated that the association between quantitative demands and skin problems decreased with higher levels of job control. See supplementary material for detailed results (Tables S1, S2, and S3).

### Additional analyses

Exposure to and experiences of psychosocial work factors may vary across occupations. The relevance of the link between the different psychosocial work factors and skin problems thus also varies. Table 5 displays occupation-specific means and standard deviations of the six psychosocial work factors measured in 2016. Of particular interest is the three psychosocial work factors that predicted skin problems in the main analyses (i.e., emotional demands, role conflict, and interpersonal conflict). Unsurprisingly, sales and service workers clearly reported highest levels of emotional demands, followed by professionals and technicians and associate professionals. Differences in role conflict and interpersonal conflict were less pronounced, but professionals reported highest levels of both, followed by managers and technicians and associate professionals.

**Table 5:** Psychosocial work factors across different occupations based on the 2016 cross-sectional sample. Occupations based on the International Standard Classification of Occupations (ISCO). Means and standard deviations in parentheses

Characteristic	Managers N = 830	Professionals N = 2,597	Technicians N = 1,282	Clerical support N = 441	Service and sales N = 1,374	Craft and related trades N = 604	Plant and machine operators N = 384	Others^*a*^ N = 321	Test of difference across occupations^*b*^
Quantitative demands	3.84 (0.82)	3.67 (0.89)	3.58 (0.92)	3.41 (0.95)	3.34 (0.98)	3.37 (0.88)	3.19 (1.06)	3.21 (0.99)	< 0.001
Job control	3.98 (0.75)	3.44 (0.72)	3.45 (0.82)	3.14 (0.86)	3.24 (0.86)	3.48 (0.80)	3.17 (0.92)	3.25 (0.90)	< 0.001
Social support	4.18 (0.89)	4.15 (0.85)	4.18 (0.88)	4.10 (0.96)	4.21 (0.88)	4.14 (0.89)	4.00 (0.95)	3.91 (1.07)	< 0.001
Emotional demands	2.19 (0.88)	2.58 (1.09)	2.34 (1.03)	2.12 (1.03)	2.79 (1.07)	1.77 (0.76)	1.94 (0.93)	1.87 (0.95)	< 0.001
Role conflict	2.13 (0.85)	2.23 (0.80)	2.09 (0.78)	1.93 (0.84)	2.03 (0.85)	2.00 (0.77)	1.96 (0.81)	2.02 (0.90)	< 0.001
Interpersonal conflict	1.83 (0.51)	1.87 (0.52)	1.81 (0.53)	1.73 (0.50)	1.77 (0.56)	1.66 (0.51)	1.69 (0.53)	1.61 (0.53)	< 0.001

^*a*^Including skilled agricultural, forestry and fishery workers, elementary occupations, and armed forces occupations

^*b*^Kruskal-Wallis rank sum test

## Discussion

The current study examined associations between psychosocial work exposures and skin problems in a large probability sample of a general working population. Emotional demands, role conflict, and interpersonal conflict emerged as significant and consistent predictors of self-reported skin problems in both cross-sectional and prospective analyses after adjusting for baseline confounders and known chemical and physical predictors of skin problems. Interpersonal conflict significantly predicted skin problems above and beyond baseline levels. Overall, our results suggest exposure to certain psychosocial stressors at work may be a risk factor for subsequent skin problems, particularly in the case of interpersonal conflict.

While our study did not examine mechanisms directly, our results are in line with those discussed in the literature on stress-related skin problems (Alexopoulos and Chrousos 2016). Potential pathophysiological mechanisms through which psychosocial work stressors lead to skin problems may include pro-inflammatory changes in neuropeptides levels, chronic activation of adrenocorticotropic-adrenal axis and skin barrier impairment (Garg et al. 2001; Fukuda, Baba and Akasaka 2015). Additionally, a work environment with an overload of psychosocial stressors may not facilitate the implementation of organizational, technical, and protective measures for prevention of skin problems at work (Alfonso et al. 2017).

The size and significance of associations differed across the different psychosocial work exposures. Interpersonal conflict is considered a leading source of stress among workers across cultures, occupations, and age groups (Spector and Bruk-Lee 2007) and is arguably more severe than the other factors we examined. Human beings are social beings (Fiske 2018), and this may make us particularly sensitive to interpersonal conflict at work (Barki and Hartwick 2004). While even episodic exposure to some amount of interpersonal conflict may be enough to trigger the body’s stress response system in a way that could lead to long-term health consequences, higher “dosages” over longer periods are probably needed for the other psychosocial work stressors to trigger the same level of psychophysiological stress responses. It is therefore not surprising that interpersonal conflict emerged as the strongest and most consistent predictor in our results. In line with potential mechanisms outlined earlier, exposure to interpersonal conflict may trigger the body’s stress response system (Nixon et al. 2011; Ganster and Rosen 2013), leading to hormonal changes that may weaken the immune system and make the skin more susceptible to inflammation and infections (Alexopoulos and Chrousos 2016; Zhang et al. 2024). Work stress resulting from experiencing interpersonal conflicts may also disrupt sleep (Yang et al. 2018) and trigger unhealthy lifestyle choices (Siegriest and Rödel 2006) that could contribute to the development or worsening of common skin conditions like atopic dermatitis, eczema, and psoriasis (Mann et al. 2023; Diotallevi et al. 2022).

Our results regarding interpersonal conflict are in line with previous studies linking low social support to skin problems in cross-sectional occupation-specific samples (Eriksson 1997; Magnavita et al. 2011). We observed a similar relationship between social support and skin problems in our study, albeit only in the 2019 sample, and social support did not emerge as a significant predictor in prospective analyses. This suggests that a certain severity in the work stressor may be needed for it to have a substantial impact on skin problems. To further elucidate this dynamic, future research could include measures of participants’ actual stress levels, physiologically or self-reported, and study how these measures covary with skin problems and exposure to stressors such as interpersonal conflicts.

In addition to interpersonal conflict, emotional demands and role conflict emerged as significant predictors of skin problems, while quantitative demands, job control, and social support generally did not. The distinction between challenge demands and hindrance demands (Van den Broeck et al. 2010) may be useful for understanding some of these results. Interpersonal conflict and role conflict are good examples of the latter type, expected to have exclusively negative effects on employee health and well-being (Van den Broeck et al. 2010). By contrast, quantitative demands such as high workload and time pressure may have both negative and positive effects, being perceived as stressful *and* stimulating at the same time (Van den Broeck et al. 2010). Thus, as a challenge demand, quantitative demands may not trigger the stress response system to such a degree that it increases the risk of skin problems in the long term. The fact that job control did not emerge as a significant predictor of skin problems is surprising considering the large knowledge body identifying job control as a strong and consistent predictor of employee health and well-being (Niedhammer, Bertrais and Witt 2021). Results regarding quantitative demands and job control run counter to some of the previous studies linking these two factors to skin problems in cross-sectional occupation-specific samples (Nomura et al. 2007; Magnavita et al. 2011).

Interactions between quantitative demands, job control, and social support was statistically non-significant, except for the two-way interaction between quantitative demands and job control in 2019. The latter finding might suggest that a combination of high quantitative demands and low job control is associated with a higher risk of experiencing skin problems relative to high demands accompanied by high job control. Yet, this should be interpreted with caution as the interaction was only significant in the 2019 sample. Thus, overall, we found limited evidence of an effect of job strain on skin problems, which again run counter to results from previous cross-sectional research (Magnavita et al. 2011).

An important practical implication of our results is that psychosocial work stressors should be considered potential risk factors for skin problems by work environment surveillance and primary and secondary prevention strategies aimed at reducing the burden of skin problems in the general working population. Additionally, our findings may be particularly relevant for occupations known to be associated with specific psychosocial work stressors, such as the high prevalence of emotional demands in client- or customer-oriented work. In some occupations, such as among healthcare workers and hairdressers, emotional demands may co-occur with other known risk factors for skin problems, such as exposure to wet work (Alfonso et al. 2015). These occupations are also among those with the highest occurrence of work-related skin problems. Future epidemiological and clinical studies assessing risk factors for work-related skin problems should therefore consider the potential influence of psychosocial work stressors and examine the impact of psychosocial and other environmental risk factors concurrently, and not only skin exposure to chemical, biological or physical factors.

### Methodological considerations

The study was based on self-report measures of psychosocial work exposures and skin problems. This means that the study first and foremost captured participants’ general experience of skin problems and investigated how these were related to participants’ own experience of their psychosocial work environment. The experience of skin problems is burdensome and may severely affect a person’s quality of life regardless of whether the condition meets clinical diagnostic criteria for specific skin diseases (Gisondi et al. 2023). Yet, it is important to note that our results may have been different if the measure of skin problems was based on a different assessment method. For instance, an 85% agreement between self-reported skin problems and expert assessment was found in a cross-sectional population-based study among young adults, using the same question on skin problems, where the disagreement was mostly due to underestimation of the work-relatedness of skin problems by the respondents (Mehlum et al. 2006; Alfonso et al. 2017). Additionally, it is important to note that our purpose with using data from the LKU-A is to conduct epidemiological surveillance of workplace conditions, wherein a symptom-based question offers several benefits. Firstly, it allows for the reporting of minor symptoms, which can help identify associations between occupational exposures and skin problems. Secondly, it has been suggested that self-reports are more likely to underestimate rather than overestimate the actual prevalence of skin conditions (Smit et al. 1992; Susitavial et al*.* 1995; Susitaival 2020). Therefore, potential bias from self-report of skin problems may lead to an underestimation of associations rather than an overestimation.

Data across two measurement occasions is a key strength in our study, enabling us to examine associations between psychosocial work exposures and skin problems prospectively. Yet, the three-year lag between timepoints is likely not ideal. While it may take some time for skin problems to manifest as a result of psychosocial work stressors, the long time lag may have resulted in effect decay over time and failure to detect the true size and significance of effects (Ford et al. 2014). Moreover, a potential healthy-worker effect may have led to weaker associations between the work exposures and skin problems at follow-up (Alfonso and Johannessen 2019).

The study used observational data to investigate associations. It is therefore limited in terms of causal interpretation. We included key demographic covariates and occupational exposures that have been found to predict skin problems in previous research (Alfonso et al. 2015). We nevertheless cannot rule out that the observed associations may be due to unmeasured confounders. Reverse causality from skin problems to the experience of psychosocial work exposures is also possible. Skin problems may severely affect a person’s quality of life, also work-related (Gisondi et al. 2023). Impaired quality of life may colour one’s perceptions of the work environment (de Lange et al*.* 2005) and those affected by skin problems could therefore have perceived and reported higher levels of psychosocial work stressors compared to those not affected. Magnavita et al. (2011) has suggested that personality characteristics (e.g., neuroticism and negative moods) may have influenced both the perception of stressors at work and the estimation of the severity of skin problems in their study among hospital workers.

For the prospective analyses, we have reported associations with and without adjusting for baseline levels of skin problems. Baseline adjustment in observational studies is a contested topic because it introduces uncertainty about what the estimated effect represents (Lydersen and Skovlund, 2021). For the current study, participants might have been exposed to a certain psychosocial work stressor at baseline, which could have already increased their risk of experiencing skin problems. Thus, baseline adjustment in this situation could represent overadjustment and lead to an underestimation of the total effect. Emotional demands and role conflict turned statistically non-significant after baseline adjustment, while interpersonal conflict predicted skin problems above and beyond baseline levels. While these results provide evidence that interpersonal conflict constitutes a risk factor for the development or worsening of skin problems, it does not exclude emotional demands and role conflict as potential risk factors.

Sampling bias is not a major concern in this study, and our findings can be generalized to the general working population of Norway, as a random sample of the Norwegian general working population was included. Although the LKU-A does not include migrant workers on short-term contracts, by 2016, these workers constituted about 3% of the working population in Norway (Tynes et al. 2018). Moreover, sampling and surveying was performed before the Covid-19 pandemic, without the influence of pandemic measures on psychosocial work exposures and chemical exposures at work due to increased hand hygiene measures.

## Conclusions

The current study examining associations between psychosocial work exposures and skin problems in a general working population suggests exposure to certain psychosocial work stressors constitutes a potential risk factor for experiencing skin problems. Interpersonal conflict emerged as the strongest and most consistent predictor, also predicting subsequent skin problems above and beyond baseline levels. Studies that further investigate these linkages therefore seem warranted. Preventive efforts to reduce the occurrence of skin problems at work should also target psychosocial work stressors and future research on risk factors for work-related skin problems should consider the effect of psychosocial work exposures.

## Supplementary Information

Below is the link to the electronic supplementary material.

Supplementary file1 (DOCX 22 KB)

## Data Availability

Data may be obtained from a third party and are not publicly available. Statistics Norway has an established policy for data sharing. Information about data requests can be accessed here: https://sikt.no/en/omrade/research-data
