# Supplementary material for: Psychosocial work exposures as risk factors for skin problems in a general working population: cross-sectional and prospective associations
Source: Int Arch Occup Environ Health. 2025 Mar 11;98(3):309–19. doi: 10.1007/s00420-025-02135-w (PMC11972187; doi:10.1007/s00420-025-02135-w)
Supplement: Supplementary file 1 — Supplementary file1 (DOCX 22 KB) [file 420_2025_2135_MOESM1_ESM.docx]

**Supplementary material**

**Table S1** Regression models testing two- and three-way interactions between quantitative demands, job control, and social support in the 2016 sample. Odds ratios (OR) and 95% confidence intervals (95% CI).

|  | Two-way interaction | | | |  | Three-way interaction | | | |
| --- | --- | --- | --- | --- | --- | --- | --- | --- | --- |
|  | Model 1 | | Model 2 | |  | Model 3 | | Model 4 | |
| Factor | OR | 95% CI | OR | 95% CI |  | OR | 95% CI | OR | 95% CI |
| Quantitative demands | 0.99 | 0.92–1.07 | 0.99 | 0.92–1.07 |  | 0.99 | 0.92–1.07 | 0.99 | 0.92–1.07 |
| Job control | 0.92 | 0.84–1.01 | 0.92 | 0.84–1.01 |  | 0.92 | 0.84–1.01 | 0.93 | 0.85–1.01 |
| Quantitative demands × Job control |  |  | 0.97 | 0.89–1.06 |  | 0.97 | 0.89–1.06 | 0.97 | 0.89–1.06 |
| Social support |  |  |  |  |  | 1.02 | 0.95–1.11 | 1.03 | 0.95–1.12 |
| Quantitative demands × Social support |  |  |  |  |  |  |  | 1.00 | 0.92–1.08 |
| Job control × Social support |  |  |  |  |  |  |  | 1.02 | 0.93–1.11 |
| Quantitative demands × Job control × Social support |  |  |  |  |  |  |  | 1.00 | 0.92–1.09 |
| *Note.* All models adjusted for age, sex, occupation, and exposure to cleaning products, water or dry indoor air in 2016. Statistically significant associations in **bold.** | | | | | | | | | |

**Table S2** Regression models testing two- and three-way interactions between quantitative demands, job control, and social support in 2019. Odds ratios (OR) and 95% confidence intervals (95% CI).

|  | Two-way interaction | | | |  | Three-way interaction | | | |
| --- | --- | --- | --- | --- | --- | --- | --- | --- | --- |
|  | Model 1 | | Model 2 | |  | Model 3 | | Model 4 | |
| Factor | OR | 95% CI | OR | 95% CI |  | OR | 95% CI | OR | 95% CI |
| Quantitative demands | 1.05 | 0.98–1.13 | 1.05 | 0.97–1.12 |  | 1.04 | 0.97–1.12 | 1.04 | 0.97–1.12 |
| Job control | 0.95 | 0.87–1.03 | 0.95 | 0.87–1.04 |  | 0.97 | 0.89–1.06 | 0.97 | 0.88–1.05 |
| Quantitative demands × Job control |  |  | **0.92** | 0.85–1.00 |  | **0.91** | 0.84–0.99 | **0.91** | 0.84–0.99 |
| Social support |  |  |  |  |  | **0.90** | 0.84–0.97 | **0.90** | 0.83–0.97 |
| Quantitative demands × Social support |  |  |  |  |  |  |  | 1.01 | 0.94–1.09 |
| Job control × Social support |  |  |  |  |  |  |  | 0.94 | 0.86–1.02 |
| Quantitative demands × Job control × Social support |  |  |  |  |  |  |  | 1.02 | 0.94–1.10 |
| *Note.* All models adjusted for age, sex, occupation, and exposure to cleaning products, water or dry indoor air in 2019. Statistically significant associations in **bold.** | | | | | | | | | |

**Table S3** Regression models testing two- and three-way interactions between quantitative demands, job control, and social support in the prospective sample. Odds ratios (OR) and 95% confidence intervals (95% CI).

|  | Two-way interaction | | | |  | Three-way interaction | | | |
| --- | --- | --- | --- | --- | --- | --- | --- | --- | --- |
|  | Model 1 | | Model 2 | |  | Model 3 | | Model 4 | |
| Factor | OR | 95% CI | OR | 95% CI |  | OR | 95% CI | OR | 95% CI |
| Quantitative demands | 0.95 | 0.85, 1.07 | 0.95 | 0.85, 1.07 |  | 0.95 | 0.84, 1.06 | 0.94 | 0.83, 1.06 |
| Job control | 0.98 | 0.85, 1.12 | 0.97 | 0.85, 1.12 |  | 0.98 | 0.86, 1.13 | 1.00 | 0.86, 1.15 |
| Quantitative demands × Job control |  |  | 0.96 | 0.84, 1.10 |  | 0.97 | 0.84, 1.11 | 0.98 | 0.85, 1.13 |
| Social support |  |  |  |  |  | 0.93 | 0.82, 1.05 | 0.94 | 0.83, 1.07 |
| Quantitative demands × Social support |  |  |  |  |  |  |  | 0.99 | 0.87, 1.13 |
| Job control × Social support |  |  |  |  |  |  |  | 1.09 | 0.94, 1.25 |
| Quantitative demands × Job control × Social support |  |  |  |  |  |  |  | 1.05 | 0.90, 1.23 |
| *Note.* All models adjusted for age, sex, occupation, and exposure to cleaning products, water or dry indoor air in 2016. Statistically significant associations in **bold.** | | | | | | | | | |
